# Effects of temporal and spatiotemporal cues on detection of dynamic road hazards

**DOI:** 10.1186/s41235-021-00348-4

**Published:** 2021-12-20

**Authors:** Benjamin Wolfe, Anna Kosovicheva, Simon Stent, Ruth Rosenholtz

**Affiliations:** 1 Department of Psychology, University of Toronto Mississauga, Mississauga, Canada; 2 Toyota Research Institute, Palo Alto, USA; 3 Department of Brain and Cognitive Sciences, Massachusetts Institute of Technology, Cambridge, USA; 4 Computer Science and Artificial Intelligence Laboratory, Massachusetts Institute of Technology, Cambridge, USA

**Keywords:** Driving, Attentional cueing, Hazard detection, Peripheral vision, Scene perception

## Abstract

While driving, dangerous situations can occur quickly, and giving drivers extra time to respond may make the road safer for everyone. Extensive research on attentional cueing in cognitive psychology has shown that targets are detected faster when preceded by a spatially valid cue, and slower when preceded by an invalid cue. However, it is unknown how these standard laboratory-based cueing effects may translate to dynamic, real-world situations like driving, where potential targets (i.e., hazardous events) are inherently more complex and variable. Observers in our study were required to correctly localize hazards in dynamic road scenes across three cue conditions (temporal, spatiotemporal valid and spatiotemporal invalid), and a no-cue baseline. All cues were presented at the first moment the hazardous situation began. Both types of valid cues reduced reaction time (by 58 and 60 ms, respectively, with no significant difference between them, a larger effect than in many classic studies). In addition, observers’ ability to accurately localize hazards dropped 11% in the spatiotemporal invalid condition, a result with dangerous implications on the road. This work demonstrates that, in spite of this added complexity, classic cueing effects persist—and may even be enhanced—for the detection of real-world hazards, and that valid cues have the potential to benefit drivers on the road.

**Supplementary Information:**

The online version contains supplementary material available at 10.1186/s41235-021-00348-4.

## Significance

While attentional cueing effects are well-established in fundamental cognitive psychology research, less is known about how these effects translate to complex, dynamic environments like the road. Understanding these effects is important, since effects that might be merely notable in the lab may have profound consequences for drivers on the road. Here, we draw on the attentional cueing literature to examine how visual cues might be used to alert drivers to emerging road hazards that would otherwise lead to a collision. We report that valid cues speed responses, while invalid cues result in a larger proportion of missed hazards, a result with severe consequences on the road. We show that these classic findings generalize to more complex situations, while emphasizing the need to experimentally extend these findings into specific applied settings to understand how they are influenced by environment and task.

## Introduction

Driving safely requires perceiving and reacting to dangerous situations or road hazards promptly to avoid a collision (Alberti et al., 2014; Crundall, 2016; Underwood et al., 2013; Wolfe et al., 2020a, b). Road hazards can take many forms, such as other drivers behaving erratically, animals entering the roadway, objects falling from vehicles, and many other unexpected events. A driver’s primary perceptual task is to acquire sufficient information about their environment in order to respond safely and promptly (Wolfe et al., 2020a, b). This is already challenging for many drivers, and is made worse by the problem of driver distraction (Strayer & Cooper, 2015; Strayer et al., 2015; Wolfe et al., 2019) which may limit drivers’ awareness of their surroundings. How can a car help a driver acquire the information they need in a timely fashion? One potential solution is to cue or alert the driver to the hazard. Research on attentional cueing in cognitive psychology has shown that, when cued to a location in space, observers will detect a subsequent object at that location faster than at other locations. However, as we discuss, these well-known results are derived from experiments with simple stimuli and little time pressure. Does this effect extend to the complex situations that drivers encounter on the road?

Cueing a driver is not a new idea; any new car on the road today will cue its driver in various ways to changes in and outside the vehicle. For example, your car might indicate an object in your blind spot, or that a tire has low air pressure, or that the engine needs a tune-up. While these cues will attract your attention, as they are designed to, they do not answer the questions at the core of this paper. Here, we asked two questions: first, do classic attentional cueing effects translate to the detection of road hazards? Although classic cueing effects have been reproduced in many studies, dynamic road scenes are highly complex, and introduce a number of additional visual and cognitive factors that might affect how these cues are processed. Second, do different cues have different consequences on drivers’ ability to respond to hazards? This is particularly timely because many new vehicles come equipped with a suite of sensors that monitor the environment to enable new driver safety features (e.g., automatic emergency braking, advanced cruise control and some limited self-driving modes) that could be used to gather information necessary to trigger these cues, if we knew them to be useful.

Attentional cueing has been extensively studied in cognitive psychology, but primarily with simple, static displays. Perhaps the best-known findings here are those of Posner and collaborators, who demonstrated that attention can be cued to peripheral locations (Posner, 1980; Posner et al., 1978). Their experiments used a simple but revealing paradigm in which observers fixated a central location flanked by two illuminated boxes to the left and right. Observers would be peripherally cued to one of these two locations prior to target presentation; if the cue and target location matched, observers were 25 ms faster to detect the target when it appeared at the cued location than they were with no cue. If they did not match, observers were slower by a similar amount, for a total range of 50 ms. These results suggest that responses are faster when correct locations are cued and slower when an incorrect location is cued.

Further work has shown that these exogenous cues, like a flashing light away from the point of fixation, are difficult to ignore, even when observers are instructed to do so (Jonides, 1981) Additionally, these reaction time effects vary depending on the interval between cue and target onset, with a maximum effect at the cued location within approximately 100–200 ms of cue presentation (Müller & Rabbitt, 1989; Nakayama & Mackeben, 1989). In contrast, endogenous cues, such as an arrow presented at fixation that points to a peripheral location, require the observer to interpret the cue and map it to a location in the world. These cues operate on a slower time course (Peterson & Gibson, 2011), with peak efficacy at a 300 ms stimulus onset asynchrony.

In addition, attentional cues can be used to indicate events in time, rather than locations in space (Correa et al., 2005; Denison et al., 2017; Nobre & Rohenkohl, 2014; Rohenkohl et al., 2011, 2014). Analogous to cues in the spatial domain, temporal cues indicate the time of an upcoming target, and can be either endogenous or exogenous (Rohenkohl et al., 2011), with comparable reaction time costs and benefits. For example, valid temporal cues produce response times that are 50 ms faster than for invalid temporal cues (Coull & Nobre, 1998). Critically, temporal cueing effects are strongest shortly after the temporal cue appears, suggesting that cueing a driver too early before a dangerous event occurs will not have an effect.

On the whole, results from these cueing studies suggest that, in principle, providing observers with spatially or temporally valid information about an upcoming road hazard could produce faster responses, and that exogenous cues may be most effective when rapid orienting is required. However, there is a considerable gap between these studies and the road environment. For one, effects like changes in reaction time or missed targets may have consequences in the world that outstrip the importance placed on them in simple laboratory studies. In addition, one cannot assume that cueing ought to function similarly regardless of setting or context, particularly given the question of saliency. Previous work has shown that cues that are distinct from their surroundings will invariably capture attention (Theeuwes, 1994, 2010), and cues superimposed on a dynamic driving scene may therefore be less effective if they are not discriminable from their surroundings. Imagine a cue that captures the driver’s attention at exactly the wrong moment, making it harder for them to shift their attention to the hazard in their environment. Such a cue, which the driver might see if the cueing system in the vehicle misidentifies and miscues something else in the scene, might make it much harder for the driver to actually notice the hazard when they need to. However, moving (Yantis & Jonides, 1984) or looming (Franconeri & Simons, 2003) cues in the periphery might be better at capturing attention. Notably, these effects have been seen when novel objects are abruptly added to dynamic environments (Karacan & Hayhoe, 2008; Shinoda et al., 2001; Yeung & Wong, 2015), suggesting that such an effect might operate in a driving context. These effects also vary with the drivers’ attentional state and level of expertise (Underwood et al., 2003); drivers are always multitasking to some degree (Boot et al., 2005) and capture is also known to be reduced under conditions of high perceptual load (Cosman & Vecera, 2010). In addition to these considerations of cue saliency, the targets (i.e., hazards) themselves are also often salient (for example, objects moving towards the driver, providing a looming cue), which may eliminate the need for a visual cue. Moreover, unlike the sudden temporal onsets seen in standard cueing paradigms, hazardous situations unfold over time, and drivers should be monitoring their environment, which may further limit the utility of any hazard cue.

Aside from these considerations in saliency and attentional capture, there are a number of other factors that may impact cueing in driving scenarios. To begin with, a hazardous situation may take some time for the driver to comprehend, and the time required to do so might mask any potential reaction time costs or benefits produced by the visual cues. For unexpected hazards, the worst-case scenario on the road, drivers must comprehend what is going on, formulate a plan and act on it very quickly, within 1500 ms (Green, 2000). Previously, we have shown that approximately 200 ms of this time can be attributed to simply acquiring enough visual information to detect the hazard, based on observers’ viewing duration thresholds in natural road video (Wolfe et al., 2020a, b). This places an upper limit on the potential reaction time benefits of these cues, and we note that an exogenous cue may only be partially interpreted in that time, limiting its potential utility on the road.

Finally, there may also be asymmetries in reaction time effects for invalid compared to valid cues, as it is difficult to anticipate how drivers might react to an invalid cue. One possibility is that an irrelevant (invalid) cue may not be very effective in the presence of an immediate, dangerous hazard, as the driver might quickly reorient and respond to the hazard. Alternatively, it is possible that effects of invalid cues, which typically slow reaction time by 25 ms in the laboratory, could be exaggerated, as a driver may take some time to think about why a particular location was cued. Unlike reaction time benefits for valid cues, there is no upper limit to reaction time costs for invalid cues, and these could have severe consequences on the road. Therefore, while many cues are implemented in vehicles already, the potential effects of invalid and valid cues are unpredictable, and the question of how they operate and what they might tell us about attention and vision in this more complex, real-world, setting is yet unexplored.

Research on driver behavior specifically has addressed some of these questions, but seldom in the larger context of attentional cueing and response facilitation. In a simulator study, Rusch and colleagues used a box superimposed over a hazard to alert drivers, and found no impact of this manipulation on how younger drivers operated the simulator, suggesting that cues might not be useful at all (Rusch et al., 2013). However, in a companion study, the same team used a similar alerting paradigm with older drivers and found some improvement in hazard avoidant behaviors (Schall et al., 2013). Neither simulator study examined questions of reaction time, nor did they use invalid cues. This is understandable with a simulator study, which does not afford experimenters time to do dozens if not hundreds of trials the way a more traditional cognitive psychology study might. More recently, Muela and colleagues examined the impact that cues might have on drivers’ awareness of proximate hazards using “a what happens next” paradigm (Muela et al., 2021). Here, participants viewed clips of road videos in which playback was abruptly stopped, and then judged what will happen next in the video, selecting from a set of options in an unspeeded task. As expected, participants were more accurate in selecting the correct option when the target (a developing hazard) was validly cued compared to when it was invalidly cued. However, it is difficult to establish the relative benefits of valid cues relative to an uncued baseline in this study, as the control condition was qualitatively different (consisting of a single hazard, while the valid and invalid conditions each contained multiple potential hazards). Together, previous work in this area has not explicitly examined reaction time differences or compared cueing effects in natural road scenes to the classic cueing literature. In principle, it is possible that cues could have a larger effect on speeded, reaction-time based tasks, compared to unspeeded tasks used in previous work. In an unspeeded task, for example, the participant may have more time to decide whether the cue was relevant or not. In addition, any possible effects of temporal cues have not been measured in these more natural videos. On the whole, work in driving on questions of cueing has just scratched the surface, and there is much work to do in this real-world case of attentional cueing.

Our goal here is to extend our understanding of cueing in a real-world situation where it might have great impact, testing its applicability in the context of hazard detection with natural road videos. As we have discussed, the effects may be small, but even small effects have an outsized impact in driving, and we cannot simply assume that cueing will behave exactly as it does in the lab in this particular context. As such, this study used valid and invalid spatiotemporal cues, drawing on Posner’s classic paradigm, as well as temporal cues, drawing on work in temporal cueing.

## Materials and methods

This study was preregistered on the Open Science Framework as “Impact of Visual Cues on Localization of Road Hazards.” The preregistration information, and all materials for this study (stimuli, experimental code, anonymized data and analysis scripts) are available from OSF (https://osf.io/xuq2f/).

### Participants

Power calculations, based on effect sizes observed in a pilot experiment (*n* = 12), indicated that a minimum sample size of 94 was required to detect a reaction time difference between the no cue and temporal cue conditions at 95% power (Cohen’s dz = 0.38; *α* = 0.05). We therefore preregistered an intention to collect data from 100 participants in this study. The final sample size (after exclusions) was 100 participants. Four participants who completed the experiment did not meet the inclusion criteria and were replaced (see “Analysis” section). Participants were recruited online through Prolific (www.prolific.co), an online recruitment platform for human participants research. Participants were required to be, by self-report, between the ages of 18–35, resident in the United States of America or the United Kingdom, licensed to drive and of either normal acuity or corrected to normal acuity. After replacing the excluded participants, the final sample was comprised of 37 male and 62 female participants, with a mean age of 27.4 (*SD* = 4.5), with demographic information not provided for one participant. Since this was an online experiment, participants’ self-report is all that is available to us for inclusion and exclusion criteria for study participation.

All participants provided informed consent prior to participation, in compliance with the Common Rule (45 CFR 46), and this study was assessed as exempt from review by MIT’s Institutional Review Board, pursuant to 45 CFR 46.101(b)(2). Participants took approximately 20 min to complete the study and were paid $6 USD for their time.

### Videos

We used a subset of videos from the Road Hazard Stimulus Set; https://osf.io/uq6pc/), along with additional forward-facing road videos recorded from dashboard cameras containing hazardous, near-collision events (including uncontrolled objects, pedestrians, and other vehicles). The videos were recorded from a variety of road settings (e.g., urban, highway) and weather and lighting conditions (see Wolfe et al., 2020a, b for further details).

For the purposes of this study, the videos were temporally annotated for the time of hazard onset, as well as where the hazard was in frame (see Wolfe et al., 2020a, b) for details). We defined hazard onset as the time point when the first visible deviation of the hazardous object from its normal state occurred (i.e., the first deviation from a non-threatening trajectory). Since we ask participants to indicate whether the hazard appears on the left or right side of the video, we selected only videos and required for which the hazard appeared solely in either the left or the right half of the video. The videos we used for spatially invalid cue trials also had a spatially annotated distractor in the uncued half. Distractors were other objects in the video (e.g., vehicles, road signs, pedestrians) that did not pose an immediate hazard to the driver. We cued objects in the spatially invalid condition out of concern that participants might more easily dismiss an invalid cue to an empty region; we leave examining this question to future work. We manually verified frame-by-frame that the hazard and distractor each remained exclusively to one side of the midline from the time of hazard onset until the end of the video, and removed any videos that did not meet these criteria.

The final stimulus set used in the experiment consisted of a total of 163 video clips at 1280 × 720 pixel resolution and 30 frames per second. The Appendix includes sample videos used in the experiment. Videos varied in duration from 2034 to 5000 ms and were all trimmed to end exactly 1000 ms after the annotated hazard onset. Therefore, across all videos, the hazard appeared unpredictably between 1034 and 4000 ms from the start of the video (For examples, see Additional files 2, 3, 4, 5, 6, 7, 8).

### Procedure

The experiment was built using PsychoPy/PsychoJS v2020.1 (Peirce et al., 2019), and hosted online on Pavlovia, based on results showing that PsychoPy/PsychoJS was the lowest-latency platform for online studies of reaction time (Bridges et al., 2020). Participants were required to complete the study on a desktop or laptop computer (i.e., the experiment was disabled on mobile and tablet devices). All videos were displayed in full screen mode on observers’ web browsers on a gray background; assuming a 24″ or 60 cm desktop monitor at 60 cm viewing distance the videos would have subtended approximately 50° horizontally. We note that our reported stimulus durations and frame numbers refer to durations in the original videos encoded at 30 frames per second, and are therefore approximate. Video playback was controlled using the *requestAnimationFrame()* function, which updated the video, if necessary, before the next screen update on the user’s device, rather than assuming a fixed interval (e.g., 16.67 ms for a 60 Hz display) across devices. This allows the program to adjust the number of frames displayed to account for differences in refresh rate, matching video playback as closely as possible across devices. Importantly, this control over video playback was implemented the same way across all our cue conditions.

**Fig. 1:**
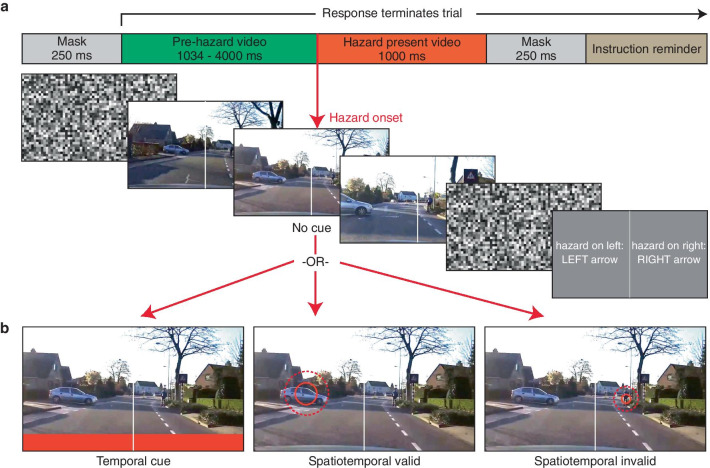
Visualization of trial sequence and cues. **a** Each trial began with a random noise mask for 250 ms, and consisted of a video that lasted between 2034 and 5034 ms. In a given video, the hazard could appear between 1034 and 4000 ms after the video began. The portion of the video containing the hazard was always 1000 ms long, and was followed by a 250 ms random noise mask. Participants were instructed to use the left and right arrow keys to indicate the lateral location of the hazard, and could respond any time after the video started. **b** The video contained either no cue or one of three cues: a temporal cue (red bar at the bottom of the video), a spatiotemporal valid cue (expanding ring superimposed on the hazard, represented by the solid red circle and the larger dashed red circle) and a spatiotemporal invalid cue (expanding ring superimposed on a nonhazardous object in the scene). The no-cue, temporal cue, and spatiotemporal conditions were blocked, and block order was randomized across participants. Within the spatiotemporal cue block, valid and invalid spatiotemporal cues were randomly interleaved and appeared with equal frequency (50% cue validity). The expanding ring spatiotemporal cue was chosen using the procedure described in the “Cue selection” section

Figure 1a shows the timeline of events for each trial. At the onset of each trial, observers were shown a mask for 250 ms, consisting of a grid of 36 × 64 squares, 20 pixels high each, with a random grayscale intensity from 0 to 255. Observers were then shown a road hazard video from one of the three cue type conditions (see *Cue Conditions* for a full description). Briefly, this consisted of either (1) no cue, (2) a temporal cue (a red horizontal bar that appeared aligned with the bottom of the video for 33 ms), or (3) a spatiotemporal cue, consisting of an expanding red ring shown for 167 ms, overlaid either on the hazard (in the valid spatiotemporal trials) or on a distractor object (in the invalid spatiotemporal trials), centered on the annotated location at the time of hazard onset (Fig. 1b). Observers were instructed to indicate the location of the hazardous event, as soon as it became hazardous to them as the driver. If the hazard was on the left half of the video, observers were instructed to press the left arrow key, and if it was on the right, to press the right arrow key. To delineate the left and right halves of the video, a white vertical line (720 pixels high and 3 pixels wide) was placed at the midline of the video for the full duration of the clip.

If the observer did not respond during the video, the clip played fully from start to end, and was followed by a second mask for 250 ms, and then a screen indicating the task instructions (see Fig. 1a). However, as the hazard always appeared 1000 ms before the end of the video, observers often responded while the video was still playing, and the instructions at the beginning of the experiment indicated that responses during the video would be necessary to perform the task quickly and accurately. If the observer responded while the video was still playing, the video ended immediately. The next trial began after the next clip finished loading, plus a 500 ms intertrial interval (ITI) with a blank gray screen.

Participants were asked to respond as quickly and accurately as they could and received visual feedback if their responses were too early (more than 500 ms prior to hazard onset, suggesting they were responding to other elements in the scene than the designated hazard) or too late (more than 2000 ms after hazard onset, suggesting that they did not notice the hazard). In either of these situations, observers were shown a black box (50% × 25% of screen height) with the text “too early!” or “too slow!” for 1000 ms following their response (however, these trials were included in the calculation of median reaction time, provided the responses were correct). Responses were only coded as correct if participants’ responses matched the location of the hazard. If the participant responded incorrectly within these time bounds, they were shown a red box with the text “incorrect” for 1000 ms.

### Cue selection

To select the visual appearance of the spatiotemporal cue, we conducted a series of five preliminary cue selection experiments (each with different set of 20 observers per experiment) comparing the effect of cue validity (invalid vs valid trials) on reaction time between pairs of candidate cue types in a within-participant design using the trial sequence illustrated in Fig. 1. There were six candidate cue types, all of which were intended to be highly visible on a variety of video backgrounds (see Table 1). As the scenes were dynamic, with the target and distractor locations changing from frame-to-frame, all the cues were intentionally selected to be brief. However, some evidence suggests that brief cues, particularly when there is little to no delay between the cue and the target, may be less effective than longer or more temporally separated cues (Lu, 2006; Matsuda & Iwasaki, 2012); the goal of the selection experiments was to find the most experimentally useful cue, rather than to determine the ideal duration for a cue used in a driving context.

**Table 1:** Table describing the six cues used in the cue selection experiments and the reasoning behind their inclusion in the set of candidate cues

Cue type	Reasoning	Description
Red bounding box	Likely highly salient as compared to the larger road environment (e.g., green plants or grey/black roadways); (Bauer et al., 1996)	Red rectangular outline shown for 33 ms (1 frame), corresponding to the annotated location, drawn with a 5 px stroke width
Static zebra-striped bounding box	Maximizes luminance contrast of the cue versus background for arbitrary background luminance; increases cue salience (Engmann et al., 2009)	Black and white frame shown for 167 ms (5 frames), corresponding to the annotated location, with adjacent concentric alternating black and white outlines (white-black-white-black at 5 px each, for a total stroke width of 20 px)
Flashing zebra-striped bounding box	Maximizes luminance contrast of the cue versus background for arbitrary background luminance, may capture attention better than a static version (Stolte & Ansorge, 2021)	As above, but the black and white elements reversed on every frame (white-to-black and black-to-white), with the cue shown for 167 ms (5 frames)
Expanding ring (spatiotemporal cue used in main experiment)	Looming stimuli are known to capture attention and may be more salient superimposed on a complex, dynamic scene (Franconeri & Simons, 2003)	Red circular outline shown for 167 ms (5 frames). The diameter of the ring on the first frame was equal to the average of half the annotated height and width of the hazard (or distractor) and increased by 40% on each consecutive video frame
Contracting ring	Some evidence suggest that motion onsets alone may capture attention and may be sufficient for a cue (Abrams & Christ, 2005)	Red circular outline shown for 167 ms (5 frames). The diameter of the ring on the *last* of the five frames was equal to the sum of the annotated height and width of the hazard (or distractor), and the diameter on each *preceding* frame was 20% larger
Flashing red dot	Used on grounds of being perhaps the easiest to implement, but likely the least salient or prone to capture attention of any cue in the set	Red filled dot on the center of the annotated hazard location (22 pixels in diameter), appeared and disappeared on alternating frames for 7 frames (4 on-frames)

**Fig. 2:**
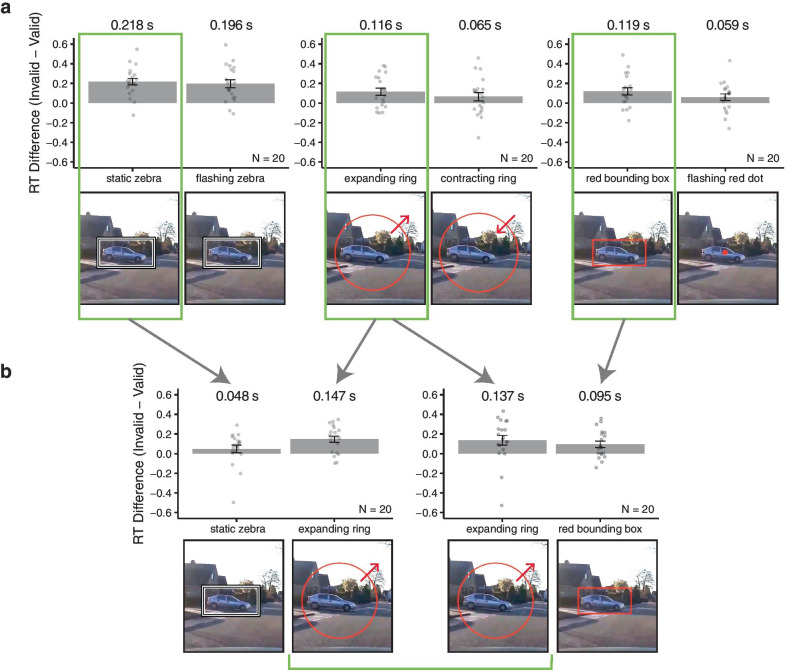
Diagram of the set of five cue selection experiments used to evaluate different cue types. There were 100 participants in total (*n* = 20 per cue selection experiment); each pair of bars represents one experiment, each with a different group of participants. The *y*-axis on each bar graph indicates the difference in reaction time (in seconds) between the invalid and valid cue conditions, with positive values indicating slower reaction times in the invalid cue condition. Note that in the main experiment, the RT difference between valid and invalid spatiotemporal cues was approximately 120 ms. Error bars indicate ± 1 SEM. **a** The first set of three experiments measured the cue validity effect for the static versus flashing zebra cues, the expanding versus contracting rings, and the red bounding box versus flashing red dot. **b** The three cue types with the larger RT difference within each paired comparison (the static zebra, the expanding ring, and the red bounding box) were used in two additional paired comparisons: static zebra versus expanding ring and expanding ring versus red bounding box. In all 5 experiments, all pairwise differences between cue types conditions were not statistically significant, but the cue with the largest invalid-to-valid RT difference within the second set of experiments—the expanding ring—was chosen for the main experiment

We first ran three experiments, each of which was a paired comparison of two cue types (Fig. 2a). We then selected, within each pair, the condition that had the larger cue validity effect (reaction time difference between invalid and valid cues) to use in a second set of experiments. The second set of experiments consisted of two additional paired comparisons using two pairs of cue type conditions (Fig. 2b). The expanding ring cue had the largest reaction time difference between valid and invalid cues in head-to-head comparisons, although it should be noted that the size of the cue-validity effect was not significantly different between any of the individual pairs of cues (*t*(19) ≤ 2.01, *p* > 0.05 for all pairs). Future work focused on the question of the best cue for a particular real-world application may want to consider factors like the contrast of the cue versus the scene as a whole (c.f., Fuller et al., 2009). Critically, this set of experiments was only meant to determine the cue with the largest effect in our particular experimental paradigm, not to determine what the best or most practical cue might be for an in-vehicle heads-up display.

Within each cue selection experiment, there were two cue type conditions (spatiotemporal valid and spatiotemporal invalid). Observers completed each of the two cue type conditions in separate blocks of trials in a random order. Each block consisted of 16 practice trials and 52 experiment trials. Within each block, there were an equal number of trials with left versus right hazards and valid versus invalid cues, which were all presented in a random order. Before starting the experiment, observers also completed 6 additional practice trials without any cues, to familiarize themselves with the videos shown. Each participant completed 142 trials in total. All other procedures were the same as those used in the main experiment (as illustrated in Fig. 1).

The process of cue type selection is illustrated in Fig. 2. Note that cue appearance may be a factor beyond the cues used in this pilot study (as discussed in Table 1), and that attentional capture is manipulated by the relative salience of the cue versus the surrounding scene (Cosman & Vecera, 2010). We chose to compare the effects of cue validity within individual pairs of cues, rather than comparing all six candidate cue types simultaneously in a within-participant design, due to the limited number of videos available.

### Cue conditions

The main experiment consisted of three conditions: a no-cue condition, a temporal cue condition and a spatiotemporal cue condition, which were blocked and presented in a random order between observers to avoid task order effects. The no cue condition simply presented the videos with no additional visual information as to the temporal onset or spatial location of the hazard. In the temporal cue condition, the temporal cue was a red bar, 1280 pixels wide and 100 pixels high that appeared aligned with the bottom of the video, overlaid on the dashboard edge, for 33 ms, at the timepoint when the hazard began in the scene (see Fig. 1b). As such, it was a 100% valid temporal cue that did not provide any spatial information about where the hazard was in the scene, only *when* it appeared. This is analogous to an alert light on the dashboard or in the driver’s field of view (similar to collision alerts or blind-spot alerts). In the spatiotemporal cue condition, the spatiotemporal cue (see Fig. 1b) was an expanding red ring (5 pixel outline) which was either overlaid on the hazard (in the valid spatiotemporal trials) or on a distractor object (in the invalid spatiotemporal trials), centered on the annotated location (see *Cue Selection* for how this cue was chosen). The ring was first visible beginning at hazard onset, and was shown for 167 ms (5 frames). The diameter of the ring on the first frame was equal to the average of half the annotated height and width of the hazard (or distractor), and increased by 40% on each consecutive video frame. Across all videos, the median cue diameter on the first frame was 43 pixels (with 95% of videos falling between 9 and 187 pixels), with a final median diameter of 165 pixels.

We note that in all videos where cues were present, the cue appeared simultaneously with hazard onset. This is a departure from many laboratory spatial attention tasks (e.g., Posner, 1980), in which the cue onset precedes the onset of the target by some interval (i.e., the stimulus onset asynchrony, or SOA). The absence of a cue-target SOA in this task is deliberately intended to simulate in-vehicle alerts that may be present in real-world driving situations, where it is not possible for the system to alert the driver before there is any visual information on the road to indicate a hazard. Accordingly, the cue was presented at the annotated time of the first departure from a normal or non-hazardous trajectory.

The no-cue block consisted of 12 practice and 30 experimental trials. The temporal cue block consisted of 12 practice and 30 experimental trials, with the temporal cue present on all trials. The spatiotemporal cue block consisted of 16 practice and 52 experimental trials (evenly divided between valid and invalid spatiotemporal cues, which were randomly interleaved. There was an equal number of left and right hazard trials within each condition, and cue side was balanced with cue validity in the spatiotemporal cue condition. The slight difference in trial number in the spatiotemporal block was due to the number of available stimulus videos with acceptable distractors, and the need to balance out the number of left and right hazards, as described below. Videos were randomly assigned to each cue condition, with the constraint that spatiotemporal-invalid videos were selected from the subset of 81 videos in which a distractor had been annotated on the opposite side of the video from the hazard. By requiring the hazard and distractor to be in opposite halves of the video image, we were able to use a left/right localization task to probe scene comprehension and hazard localization, rather than simple detection. However, doing so did limit the total number of videos available from our existing stimulus set, since not all hazards satisfy this requirement (e.g., many occur directly ahead of the driver). However, all hazards were exclusively in the left or right half of the scene to avoid confusion on the localization task. In the trials with invalid spatiotemporal cues, the cued distractor item (e.g., another vehicle) was always in the opposite half of the scene. To provide observers with some examples of videos they would see in each block, they completed an initial set of 8 practice trials in the no-cue condition before starting the first block. In total, the experiment consisted of 160 trials, and took participants approximately 20 min to finish. Observers were shown a horizontal bar at the top of the display indicating their progress through the experiment and were given opportunities to take breaks between blocks.

Finally, one potential concern with the constraints on video randomization in the spatiotemporal-invalid cue condition is that differences between videos used in the conditions could account for the reaction time and accuracy effects we report in Figs. 1 and 2. We therefore separately reanalyzed the reaction time and accuracy data using a subset of the 81 videos used in this condition, for both the main experiment, and for the pilot data (using the Expanding Ring condition used in the present study). These data are shown in Additional file 1: Figures S1 and S2 and described in the “Discussion” section.

### Analysis

Data from each participant in the main experiment were evaluated relative to three preregistered criteria before inclusion in the final dataset for this study. First, participants were required to perform the left/right hazard localization task significantly above chance across all trials, as determined by a binomial test. Second, participants were additionally required to attain at least 50% accuracy individually in each of the four possible trial types (no cue, temporal cue, valid spatiotemporal cue and invalid spatiotemporal cue). For example, a participant who was significantly above chance across all trials, but did not reach 50% accuracy in the spatiotemporal-invalid condition would be excluded. Third, a participant’s median reaction time, across all trials relative to hazard onset, must have been between − 500 and 2000 ms. Note that participants were able to respond at any time the video was playing, so median reaction times may reflect their perception of hazards in the scene, rather than the point at which hazards are annotated in the stimulus videos. This is likely more reflective of real-world behavior, as alert drivers may be able to notice evolving hazards before they become immediately dangerous. Four participants were replaced for failing to reach 50% accuracy in one of the four conditions, according to our inclusion criteria. All participants had median reaction times within the specified range, with median reaction time for each participant ranging from 176 to 1716 ms (mean: 533 ms, SD: 243 ms).

**Fig. 3:**
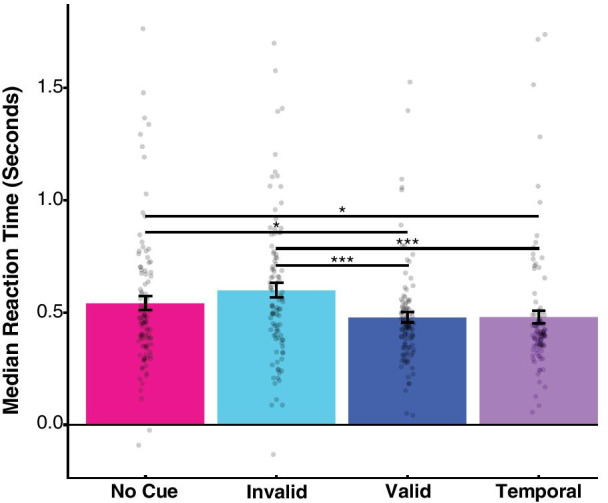
Reaction time results. Each individual dot represents one participant’s median reaction time for that condition, and bars represent the group mean within the corresponding condition. Mean reaction time in the absence of a cue was 542 ms (magenta bar); invalid spatiotemporal cue (cyan bar) was 600 ms (+ 58 ms vs no cue), valid spatiotemporal cue (navy bar) was 479 ms (− 63 ms) and temporal cue only (violet bar) was 481 ms (− 61 ms). A single asterisk represents *p* values < 0.05; three asterisks represent *p* values < .0001. Error bars are standard error of the mean

Reaction time analyses were only performed on trials where participants responded correctly, per our preregistered analysis plan. In addition, we identified four videos with excessively fast median reaction times across all observers (less than − 500 ms relative to annotated hazard onset), indicating that observers were not reliably responding to the annotated hazard (i.e., a hazardous event was reliably detected prior to the annotation). Trials that included these videos were removed from the analysis. For the included trials, we calculated each participant’s median reaction time within each condition, then calculated the mean across participants in the group data (Fig. 3). The effect of cue type on reaction time and performance was analyzed with separate one-way repeated-measures ANOVAs (with four levels each: no cue, temporal cue, valid spatiotemporal cue and invalid spatiotemporal cue), using the Greenhouse–Geisser correction for sphericity.

## Results

### Reaction time results

We observed a significant main effect of cue type in our reaction time data *F*(2.76, 272.91) = 12.84, *p* < 0.0001, *h*_p_^2^ = 0.115. To compare reaction times between conditions, we report all pairwise contrasts using the Tukey method for multiple comparisons, with adjusted *p* values (compared against *α* = 0.05). Treating our no-cue condition as a baseline (mean, 542 ms, SEM, 31 ms), we found shorter reaction times in the spatiotemporal valid cue condition (63 ms faster than baseline; mean, 479 ms, SEM, 23 ms; *t*(297) = 2.78, *p* = 0.03, *d* = 0.22) and in the temporal cue condition (61 ms faster than baseline; mean, 481 ms, SEM, 28 ms; *t*(297) = 2.71, *p* = 0.036, *d* = 0.22), indicating that participants were able to detect hazards more quickly in the videos given either a valid spatiotemporal cue or a temporal cue. Note that, using Posner (1980) as a point of comparison, which reported valid peripheral cues speeding reaction time in a detection task by approximately 25 ms, our effects are more than twice as large. However, there was no significant increase in reaction time in the spatiotemporal invalid condition compared to the baseline (difference of 58 ms; mean, 600 ms, SEM, 32 ms; t(297) = 2.54, *p* = 0.057, *d* = 0.20).

Additionally, we found significant differences between the spatiotemporal valid and invalid conditions (*t*(297) = 5.31, *p* < 0.0001, *d* = 0.42) and between the spatiotemporal invalid and temporal conditions (*t*(297) = 5.24, *p* < 0.0001, *d* = 0.42). We found no significant difference in mean reaction time between the spatiotemporal valid and the temporal conditions (*t*(297) = 0.07, *p* = 0.99, *d* = 0.01), suggesting a similar reaction time benefit of each of these cues. Given the similarity in reaction times between the temporal and valid spatiotemporal conditions, we additionally tested for the absence of a difference in reaction times between these conditions using a Bayesian t-test, an analysis that was not in the original pre-registration. The Bayes Factor of the alternative hypothesis (H1) against the null (H0), calculated using the Jeffrey–Zellner–Siow prior, indicated moderate evidence in favor of the null hypothesis, BF_10_ = 0.11.

### Accuracy results

Similar to our reaction time data, we observed a main effect of cue type in our accuracy data *F*(2.49,246.07) = 53.707, *p* < 0.0001, *h*_p_^2^ = 0.352. Again, we report the results of pairwise comparisons using the Tukey method, with adjusted *p* values. Using the no-cue condition as a point of reference (where the mean percentage of correct responses was 87.1%, SEM, 0.8%), we found similar proportions of correct responses to our no-cue baseline in the spatiotemporal valid condition (1.1% higher than baseline; mean, 88.2%, SEM, 0.8%; *t*(297) = 0.99, *p* = 0.76, *d* = 0.12) and the temporal cue condition (1.9% lower than baseline; mean, 85.2%, SEM, 0.7%; *t*(297) = 1.82, *p* = 0.27, *d* = 0.22). The absence of a difference between the no-cue and spatiotemporal valid conditions was supported by a Bayesian *t* test, indicating that participants’ accuracy was not impacted by this cue, BF_10_ = 0.21, while the comparison between the no-cue and temporal cue condition was inconclusive, BF_10_ = 1.20.

**Fig. 4:**
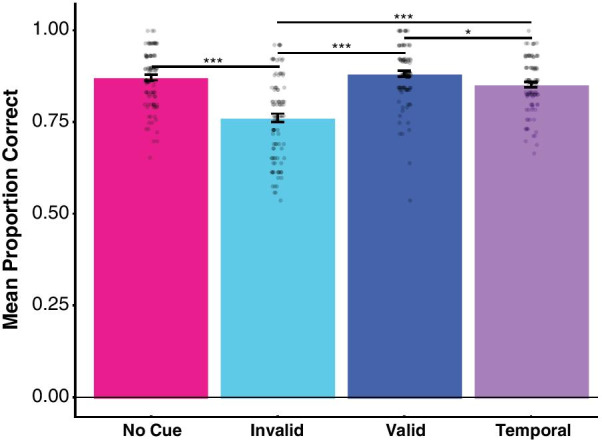
Mean proportion correct. Each individual dot represents one participant’s mean proportion correct for a given condition. Mean proportion correct in the absence of a cue (no-cue, magenta bar) was 87.1%; invalid spatiotemporal cue (cyan bar), 76.1% (− 11% vs no cue), valid spatiotemporal cue (navy bar), 88.2% (+ 1.1%) and with a temporal cue (violet bar) 85.2% (− 1.9%). A single asterisk represents *p* values < 0.05; three asterisks represent *p* values < .0001. Error bars are standard error of the mean

Compared to the no-cue baseline, observers were considerably less accurate in the spatiotemporal invalid condition (11% lower than baseline; mean, 76.1%, SEM, 1.1%; *t*(297) = 10.38, *p* < 0.0001, *d* = 1.28), in line with the increased reaction times in this condition. Observers were also significantly less accurate in the invalid compared to valid spatiotemporal cue conditions (*t*(297) = 11.37, *p* < 0.0001, *d* = 1.40), and in the spatiotemporal invalid compared to the temporal cue condition (*t*(297) = 8.56, *p* < 0.0001, *d* = 1.05). Finally, accuracy was higher in the spatiotemporal valid condition compared to the temporal cue condition (*t*(297) = 2.81, *p* = 0.027, *d* = 0.35), although the absolute difference in accuracy here is low (~ 3%), compared to the differences observed between the spatiotemporal invalid and other cues (− 11% versus baseline) (Fig. 4).

## Discussion

In this study, we asked whether well-established effects of spatial and temporal cueing translate to driving, where spatial and temporal cues were used to alert drivers to proximate hazards that required a prompt response to avoid a collision. Unlike the stimuli used in many classic cueing tasks, dynamic road scenes are inherently complex and variable, where differences in saliency, attentional capture, and scene comprehension may limit the applicability of standard cueing findings. Despite these differences, we not only replicated classic results with spatiotemporal cues, but observed somewhat larger effects than those suggested by prior findings. We showed that a valid spatiotemporal cue significantly speeded reaction times in our hazard detection task by 63 ms relative to an uncued baseline, an effect more than twice the size of the cueing effect reported by Posner (1980), and the effects seen in a meta-analysis of cueing results (Samuel & Kat, 2003), approximately 25 ms. In addition, we showed that a transient temporal cue, visible for only one frame, speeded reaction times similarly to the valid spatiotemporal cue (61 ms faster than baseline). Together, these results suggest that despite the large differences between our studies and those in the attentional cueing literature, cueing effects exist in dynamic natural environment and cues may have the potential to speed driver responses on the road.

A critical difference between our study and many studies of attentional cueing is that our task required participants to understand the dynamic road scene they were watching. They had to not only use the cue, but in order to perform the localization (left/right discrimination) task accurately, they had to understand *what* was being cued and to determine *whether or not the cued object was dangerous*. This is a significant departure from many cueing paradigms, which use simple stimulus onset detection (Posner & Cohen, 1984) or other simple visual tasks (reviewed in (Theeuwes, 2010) rather than requiring participants to understand the scene as a whole. As a result, dismissing an invalid spatiotemporal cue may have been particularly difficult for our participants, as they would need to understand that the cued object was not a hazard, and, subsequently, identify the actual hazard to be confident that they were making the correct response. The additional degree of comprehension required for this task could account for some of the differences we see from standard cueing results. Moreover, the difference in reaction time for invalid compared to valid spatiotemporal cues (121 ms) is significant and likely has real-world consequences, since 121 ms on the highway (moving at 110 kph/68 mph) is 3.7 m of travel, or nearly a full car length.

Why might we see such a large reaction time benefit for valid cues? One possible explanation for this large effect is that presenting a cue at just the right moment may help the driver come to a decision about the hazard, if it is present. A key difference in our study is that hazards evolve over time on the road, unlike the abruptly appearing stimuli common in studies of attentional cueing. If a driver is accumulating evidence towards a hazard/no hazard decision, a valid cue at the right time might speed their responses, whereas an invalid cue might slow this process. Given our results with temporal cues, which also speeded responses in our task, it is likely that cue timing is critical here. However, conclusively demonstrating that cueing effects in driving are larger than those in more fundamental studies would require within-subjects comparisons across tasks, that is, having the same participants do our task and a set of more conventional cueing tasks. This is particularly notable, because we might expect the cues we used to be less salient superimposed on videos of road scenes and the hazards themselves might have been more salient than their surroundings, both of which might have been expected to reduce any cueing effect.

In addition, there are other key differences between our experiment and classic cueing studies. Our observers freely viewed the videos used in the study, rather than maintaining fixation at a designated location at the moment the cue was presented. While laboratory studies of attentional cueing often enforce fixation, it was not appropriate to do so here, since drivers do not habitually maintain fixation at a single location while the scene moves around them (Underwood et al., 2005). In previous work, where observers monitored a road scene for events directly ahead of them while maintaining fixation at a specific off-road location, we showed that observers were still able to detect events on the road ahead with peripheral vision (Wolfe et al., 2019) regardless of whether or not they were distracted. However, they responded 200–300 ms slower than when they were looking at the road ahead, depending on target eccentricity from fixation. Since cues might be most useful when drivers are distracted and looking away from the road, future experiments might constrain participants’ gaze behavior and assess whether cues might help alleviate some of the penalties from exclusively peripheral monitoring of the road ahead.

Another consideration is the length of the delay between the cue and the stimulus, which is commonly manipulated in many cueing experiments (stimulus onset asynchronies). We presented all of our cues at the time the hazard began, as this provided our observers with the maximum time to use the information from the cue. While it would be ideal, on the road, to cue the driver before they can perceive a hazard, there is no principled way to determine what SOA would be appropriate, and delaying the cue based on how long the vehicle itself might take to assess hazards and come to its own decision is similarly difficult. However, as the computer vision algorithms which would enable such a cueing system evolve and improve, it may prove fruitful to revisit these questions, particularly in comparison to human behavior, since the goal of driver assistance systems is to augment the driver’s own capabilities. For that matter, there are presently challenges to using cues co-located with the hazard in cars, since they would need to be projected at a specific location on the windshield in a way that made them visible in any lighting environment. A purely temporal cue might, therefore, be a more practical solution in a vehicle, although both are potentially revealing to test.

Finally, moving away from reaction time effects to effects on hazard detection performance, the consequences of these cues in a driving application become exceptionally clear. While there was little difference in hazard detection performance between our baseline, valid spatiotemporal and temporal cue conditions (ranging from 85 to 88% correct), our observers incorrectly localized 11% more hazards in the invalid spatiotemporal condition. Although some laboratory studies of attentional cueing have shown similar effects on accuracy (Henderson & Macquistan, 1993; Luck & Thomas, 1999; Müller & Rabbitt, 1989), this difference in error rate would have vast consequences on the road. While our observers were not perfect in localizing hazards, this decrease from their baseline, suggests just how dangerous the wrong cue at the wrong time might be, and why we should not expect results with similar paradigms but simpler stimuli to tell us everything we need to know. We also observed this large decrease in accuracy for invalid cues in a control analysis, in which we analyzed a subset of videos matched across all conditions in the study (Additional file 1: Figure S1). However, any possible increments in reaction time for invalid cues were less conclusive, as this difference was not significant in the main study or in the same subset analysis. Further work would be necessary to determine the nature of any reaction time penalties for invalid cues, as this may depend on the identity of the cued distractor (for example, an invalid cue over a pedestrian or vehicle may produce a different reaction time penalty than one over a static background).

Overall, our results suggest that many of the well-established results in attentional cueing translate to driving, despite the large differences in stimuli—between illuminated boxes on an otherwise blank screen to pedestrians stepping into the road in a complex urban street scene. Future work at the intersection of attentional cueing and driving has the potential to inform our understanding of how we orient to stimuli in dynamic scenes, as well as how in-vehicle alerts might be used to make roads safer for everyone. For example, we demonstrate here that valid cues facilitate the speed with which a participant can accurately indicate the side of the hazard, but further work would be necessary to establish whether cues benefit speeded decisions about the most appropriate evasive maneuver. This may be the case if accurate hazard localization is the first step in the process of planning an evasive maneuver (i.e., the driver first identifies where the hazard is, and based on this information, decides what to do). Other remaining questions relate to the costs and benefits of different cue types. While we showed that temporal cues can be effective, future work might investigate whether there are any reaction time penalties associated with invalid temporal cues, that either do not cue a hazard at all or appear too early or too late to be of any use. Similarly, one could manipulate spatiotemporal cue timing based on what might happen on the road.

Further work might also investigate how these cues might interact with the attentional state of the driver. Would these cues be most useful when drivers are multitasking, rather than just focusing on detecting hazards? If drivers are relying exclusively on peripheral vision, as they would have to when looking away from the road, might the question of salience become even more important? Along these lines, we might observe even larger costs and benefits when drivers are distracted and looking away from the road. Furthermore, imperfect cueing, which may easily occur in the world when a cueing system is imperfect, is likely to magnify these effects, since the driver or user will not be able to trust it. The question of cue reliability, therefore, has disturbing implications for the use of cues in vehicles, as well as in other cases which might use similar approaches (e.g., aiding radiologists in reading scans; what happens if the radiologist gets a wrong cue and misses a cancer?). In all of these cases, such a system might be both a boon and a curse to people who use it, potentially helping in some circumstances while leading users astray in others. A driver, or a radiologist, might become too dependent on the cueing technology, trusting it over their own abilities, with consequences for missing hazards (or abnormalities). That being said, this study provides a first step towards a more complete understanding of how attentional cueing might work for drivers and in other applied settings and it suggests that these effects are worth investigating in real-world contexts. In the particular context of driving, our work here may help to point automakers towards solutions that may make roads safer for everyone, and suggest a need for a wider body of work at the intersection of attentional cueing and application.

## Conclusion

Our results indicate that attentional cueing effects exist in dynamic road scenes, in spite of the considerable differences between them and the simple displays often used to study attentional cueing in the laboratory. However, there are key nuances that emerge when cueing drivers that preclude simply assuming that results with simple stimuli will be identical in driving. We find that valid spatiotemporal cues and temporal cues speed reaction time when detecting hazards in clips of road video and invalid spatiotemporal cues also reduce hazard localization accuracy. These results have implications for vehicle design, suggesting that cueing the driver to a dangerous situation may buy them precious time to respond, and that temporal cues, when well-timed, may be adequate to drivers’ informational needs. Our approach, drawing on the attentional cueing literature and applying it to a real-world context, has revealed key nuances in how cues impact our perception of the world and has pointed the way towards how cars may better support their drivers.

## Supplementary Information

**Additional file 1**. **Figure S1**, subset analysis (invalid cue videos only); **Figure S2**, reaction time analysis of cue comparison data, using only invalid cue videos.

**Additional file 2**. Example video used in the study; no cue condition.

**Additional file 3**. Example video used in the study, temporal cue condition.

**Additional file 4**. Example video used in the study, spatiotemporal valid cue condition.

**Additional file 5**. Example video used in the study, spatiotemporal invalid cue condition.

**Additional file 6**. Additional examples of annotated hazards, all shown in the spatiotemporal-valid cue condition. Hazards could appear in the left or right half of the video, and included animals (a).

**Additional file 7**. Additional examples of annotated hazards, all shown in the spatiotemporal-valid cue condition. Hazards could appear in the left or right half of the video, and other vehicles (b).

**Additional file 8**. Additional examples of annotated hazards, all shown in the spatiotemporal-valid cue condition. Hazards could appear in the left or right half of the video, and pedestrians (c).

## Data Availability

This study was preregistered on the Open Science Framework as “Impact of Visual Cues on Localization of Road Hazards.” The preregistration information, and all materials for this study (stimuli, experimental code, anonymized data and analysis scripts) are available from OSF (https://osf.io/xuq2f/).
