# Supplementary material for: Effects of temporal and spatiotemporal cues on detection of dynamic road hazards
Source: Cogn Res Princ Implic. 2021 Dec 20;6:80. doi: 10.1186/s41235-021-00348-4 (PMC8688617; doi:10.1186/s41235-021-00348-4)
Supplement: Supplementary file 1 — Additional file 1. Figure S1, subset analysis (invalid cue videos only); Figure S2, reaction time analysis of cue comparison data, using only invalid cue videos. [file 41235_2021_348_MOESM1_ESM.pdf]

Supplemental Figures: Effects of Temporal and Spatiotemporal Cues on Detection of Dynamic Road Hazards

Benjamin Wolfe, Anna Kosovicheva, Simon Stent, Ruth Rosenholtz

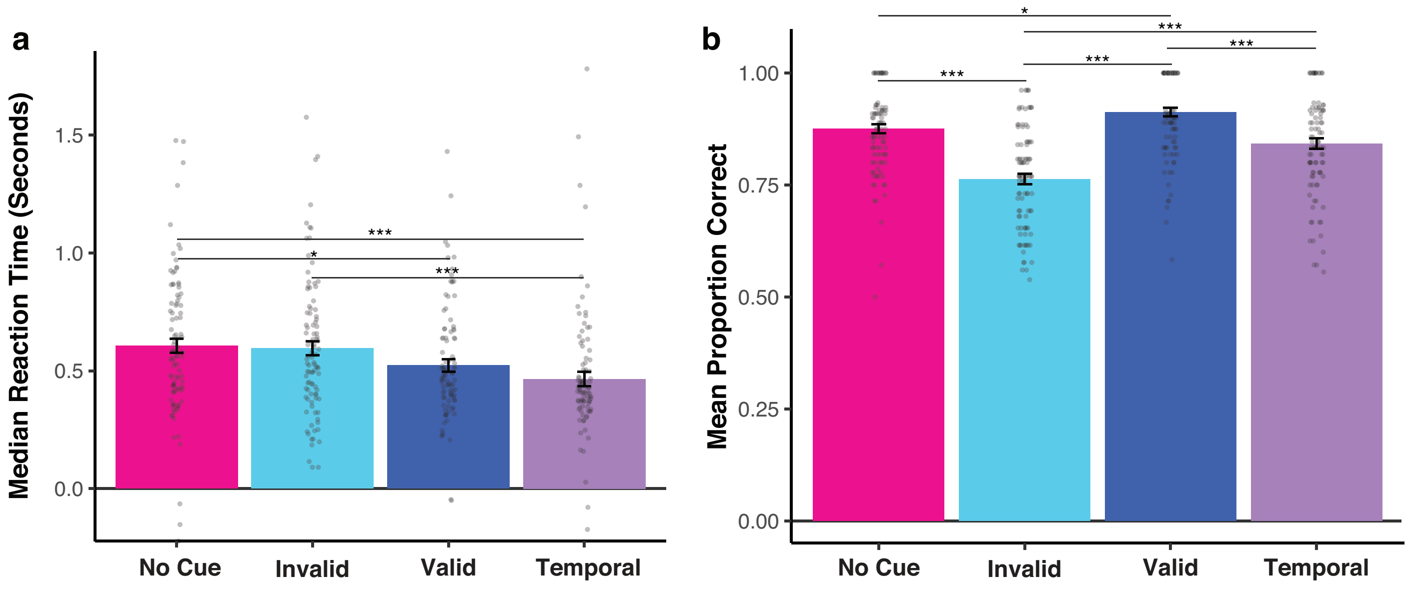

Figure S1. Analyses shown in Figures 3 and 4 using a subset of trials in the main experiment, consisting only of the 81 videos that were used in the invalid cue condition. (a) Mean reaction time in the absence of a cue was 606 ms (magenta bar); invalid spatiotemporal cue (cyan bar) was 595 ms (-11 ms vs no cue), valid spatiotemporal cue (navy bar) was 523 ms (-83 ms) and temporal cue only (violet bar) was 465 ms (-141 ms). (b) Mean proportion correct in the absence of a cue (no-cue, magenta bar) was 87.6%; invalid spatiotemporal cue (cyan bar), 76.3% (-11.3% vs no cue), valid spatiotemporal cue (navy bar), 91.3% (+3.7%) and with a temporal cue (violet bar) 84.2% (-3.4%). A single asterisk represents p-values < 0.05; three asterisks represent p-values < .0001. Error bars are standard error of the mean.

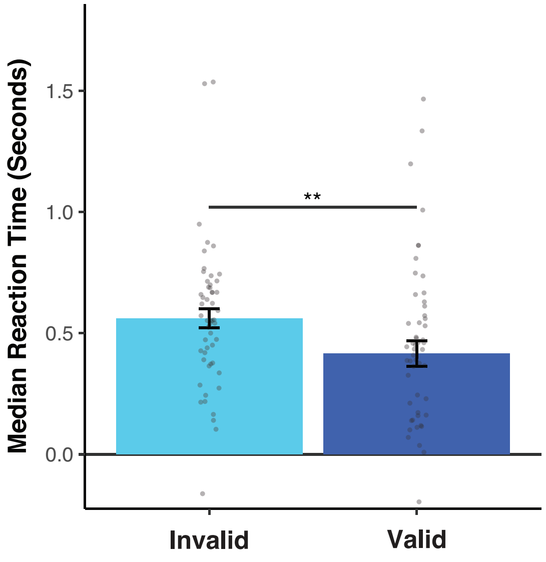

Figure S2. Analysis of reaction times from the cue selection pilot study (shown in Figure 2), using only the subset of 81 videos that were used in the invalid cue condition. Reaction times are shown separately for the invalid and valid cue conditions, aggregated across the expanding ring cue condition in the three

cue selection experiments that used this cue (the same cue as used in the main study). Note that the other conditions (no cue, temporal cue) were not tested in this pilot experiment. Asterisks denote a significantly faster reaction times in the valid cue condition (416 ms) compared to the invalid cue condition (561 ms), a difference of 145 ms, $t(53) = 2.96$, $p = .005$, $d = .40$. Error bars are standard error of the mean.
